# Nurses’ accuracy and self-perceived ability using the Emergency Severity Index triage tool: a cross-sectional study in four Swiss hospitals

**DOI:** 10.1186/s13049-015-0142-y

**Published:** 2015-08-28

**Authors:** Karin Jordi, Florian Grossmann, Gary M. Gaddis, Eva Cignacco, Kris Denhaerynck, René Schwendimann, Christian H. Nickel

**Affiliations:** Institute of Nursing Science, University of Basel, Basel, Switzerland; Department of practice development, Hospitals of Canton Solothurn, Olten, Switzerland; Emergency Department, University Hospital Basel, Basel, Switzerland; St. Luke’s Hospital of Kansas City and the University of Missouri-Kansas City School of Medicine, Kansas City, MO USA

## Abstract

**Background:**

The Emergency Severity Index (ESI) is an English language emergency department patient triage tool. After translation, it has been adapted for use to triage patients in growing numbers of emergency departments in non-English-speaking countries. Few reports of the proficiency of triage nurses to score an ESI exist. We sought to determine accuracy, inter-rater reliability, and subjective confidence of triage nurses at four hospitals to determine an ESI from standardized ESI scenarios.

**Methods:**

Triage nurses assigned an ESI score to each of 30 standard ESI (ESI Implementation Handbook Version 4) translated teaching case scenarios. Accuracy and Inter-rater reliability (Krippendorff’s alpha) of the ESI scoring was measured. Nurses’ subjective confidence applying the ESI algorithm was obtained by a Likert scale.

**Results:**

Sixty-nine nurses from four EDs participated in the study. They scored 59.6 % of the case scenarios correctly. Inter-rater reliability was 0.78 (Krippendorff’s alpha). Most (54/69, 78 %) felt confident in their ability to apply the ESI.

**Conclusions:**

Low accuracy of ESI score assignment was observed when nurses scored an ESI for 30 standard written case scenarios, translated into nurses’ native language, despite a good inter-rater reliability and high nurse confidence in their ability to apply the ESI. Although feasible, using standard written case scenarios to determine ESI triage scoring effectiveness may not be the optimum means to rate nurses’ triage skills.

## Introduction

Triage is a sorting process by which Emergency Department (ED) clinicians prioritize patients for care at their time of arrival to an ED. Triage considers patients’ level of acuity and potential for threat to life due to their condition, as well as patients’ anticipated resource need. It is therefore a vulnerable event in the patients’ evaluation. Various standardized scoring rubrics exist to sort and rank patients. The Emergency Severity Index (ESI) is one such rubric [1]. It is a reliable and valid [2–4] five-level instrument. It is the most widely utilized triage scoring system in the United States [5] and is becoming more widely deployed in non-English speaking countries [6].

All clinicians who apply the Emergency Severity Index (ESI) in clinical practice must be competent to make rapid and accurate decisions with confidence, as informed by the decision points of the algorithm. Those decision points assess for a potential requirement for life-saving interventions, whether the patient cannot safely wait to be seen by a physician and the appropriate number of resources [4]. As these triage decisions directly influence patient outcomes, and as the ESI has become more widely used in non-English speaking countries, an evaluation of the education, training, implementation and maintenance of accurate triage acuity rating skills by triage nurses who employ the ESI in non-English speaking countries deserves special attention.

Written case scenarios that typify patients who might present to an ED and which are provided in the ESI implementation handbook [1] (Version 4) can be used as a tool to assess ED nurses’ uptake of learning outcomes and competence in triage decision making. Previously, different and more labor-intensive methods for triage training, such as human patient simulation and algorithm-based courses have been used [1, 7–12].

To our knowledge, no studies about the inter-rater reliability and accuracy of triage decisions of German-speaking nurses, using the written scenarios provided in the ESI implementation handbook [1], from more than two different hospital settings have been reported.

Objectives of this study included: a) to examine the accuracy of triage acuity ratings by ED triage nurses, who derived these ratings from 30 standard written test case scenarios using the ESI algorithm, with questions provided in the ESI implementation handbook translated into German; b) to verify the interrater reliability of ESI scoring across different hospital and training settings, and c) to assess nurses’ subjective confidence in applying the ESI.

## Materials and methods

### Study design, sample and setting

This cross-sectional, multicenter study was conducted in the EDs of four hospitals in the German-speaking part of Switzerland. The participating hospitals included: two tertiary care hospitals (Hospital A and Hospital C), and two secondary care hospitals (Hospital B and Hospital D). In the cities in which the tertiary hospitals are located, most children younger than 16 years of age are treated at the local children’s hospitals Children with head trauma or major trauma are treated in the adult ED. In hospital B and in hospital D children are treated only for minor diseases and injuries. All of the participating hospital sites implemented emergency nursing triage with the ESI (Version 4) between 2008 and 2011. Characteristics of the participating hospitals, the training and qualification requirements for triage nurses, the year of introduction of ESI triage, the duration of nurses’ experience with ESI, and ongoing ESI training is displayed in Table 1. Of note, all participating study centers comply with the recommendations of the ESI handbook [1]. The study was approved by the local ethical boards of the respective cantons (identifiers: EKBB 250/11, KEK-ZH 2011–0392, EK AG 2011/058).

**Table 1:** Characteristics of the participating emergency departments and hospitals

	Hospital A	Hospital B	Hospital C	Hospital D
Number of beds	700	300	852	235
Level of care	urban, tertiary-care hospital	secondary care hospital	urban, tertiary-care hospital	secondary care hospital
Annual ED census	43,000	20,600	36,000	16,900
Triage team	17 nurses	19 nurses	53 nurses	12 nurses
Qualification requirements for triage nurses	Postgraduate emergency nursing education^a^ OR many years of professional ED experience AND triage training (see below)	Nurses with or without postgraduate emergency nursing education^a^, at least six month experience in emergency nursing AND triage training (see below)	Postgraduate emergency nursing education^a^ OR Many years of professional ED experience, more than 4 years of experience in acute nursing AND triage training (see below)	Postgraduate emergency nursing education^a^ AND triage training (see below)
Introduction of the ESI	2008	2011	2010	2009
Experience with the ESI in daily practice, months (min-max)	32 (2–48)	11 (7–11)	12 (4–14)	23 (10–30)
Training	Three hours of training in ESI application, with supervision by an experienced ESI user, for each nurse’s first day of implementation of the ESI tool. Four times annually, refreshing the knowledge in team meetings by discussing cases.	Four hours of training in ESI application, with supervision by an experienced ESI user, for each nurse’s first day of implementation of the ESI tool	Four hours of training in ESI application, and, once a month, participation in a triage workshop.	Three hours of training in ESI application, with supervision by an experienced ESI user, for each nurse’s first day of implementation of the ESI tool

Characteristics of the participating hospitals, the training and qualification requirements for triage nurses, year of introduction of ESI triage, the duration of nurses’ experience with the ESI, and ongoing ESI training is displayed

^a^Comprises similar postgraduate education, such as intensive care or anesthesia nursing

Triage nurses were eligible to participate if they had previously undergone between 3 and 4 h of training in ESI (Version 4) application when the ESI was implemented at their institution. Prior to initiating the study, no additional training or instruction was provided. Excluded from the study were part-time RNs who worked less than 16.8 h per week.

### The Emergency Severity Index (ESI)

The ESI is a five-level ED triage instrument [1]. Level 1 indicates the most urgent and level 5 the least urgent level. The ESI algorithm, which facilitates the determination of patient acuity, has four decision points. The first two decision points indicate whether the patient is in need of an immediate life-saving intervention (decision point A for ESI level 1) or should not wait to be seen by an ED care provider (decision point B, ESI level 2). At decision point C the number of diagnostic and treatment resources needed is anticipated, such as radiographs, laboratory studies, electrocardiogram, IV fluids, IV medications, specialist consultation or procedures, (no resources needed: ESI level 5, one resource needed: ESI level 4, two or more resources: proceed to decision point D). At decision point D the triage nurse evaluates the patient’s vital signs (normal vital signs: indicates ESI level 3, abnormal vital signs indicate ESI level 2 must be considered).

### German-language materials

The latest version of the ESI, available at the time of the study (Version 4), and the 30 case scenarios, were translated into the German language using a guideline based approach. Recommendations for translation of instruments as developed by the International Society for Pharmacoeconomics and Outcome Research (ISPOR) Task Force for Translation and Cultural Adaptation (which is similar to the procedure suggested by Gjersing et al. [13, 14]) were utilized. Similar processes were followed in translating the ESI instrument into the German language [6]. This involved two forward translations, the synthesis of an integrated forward translation, two backward translations, comparison of the translated version with the original, and a review of clarity of expression of the translation. These processes were completed independently by an advanced practice nurse (FFG) and an attending emergency physician (CHN) for the forward translation. The synthesis was completed by the research team leading to one integrated forward translation. Two native-English-speaking Nurse Practitioners independently performed a backward translation. The backward translation was compared with the original version by the research team. The final translated patient scenarios were reviewed by two instructors for postgraduate emergency education and an ED ward nurse for applicability. This translation was known to provide very good inter-rater reliability and validity when applied to all ED comers [6] and also older patients [7].

The ESI implementation handbook (Version 4, 2005) [1] contains 30 written case scenarios for which the authors have determined the correct ESI classification.

The handbook contains six ESI level 1 cases, six ESI level 2 cases, seven ESI level 3 cases, six ESI level 4 cases and five ESI level 5 cases. Children are represented in six of the 30 case scenarios. Nine of the 24 adult cases include trauma patients, whereas 15 describe non-trauma patients.

### Measurements

#### Accuracy

An accurate triage rating was defined as a participant’s response matching with the correct response, as defined by the ESI implementation handbook [1].

#### Inter-rater reliability

We defined inter-rater reliability as the degree of concordance of the participants in applying the ESI.

#### Confidence in applying the ESI

Confidence was defined as the subjective level of confidence (from very confident to very unconfident via a five-level Likert scale) as expressed by nurses using the ESI both in clinical practice and in performing the case scenarios.

### Data collection

The survey data were collected between September 1, 2011, and February 29, 2012, during team meetings. Prior to the study, all eligible participants received written information about the study from the principal investigator. The information sheet for the participants consisted of the study’s aims and the data collection procedure. Blinding of the investigators was assured to permit nurses’ answers to remain anonymous. Non-participation did not result in any penalties. All participants signed an informed consent form.

Study participants completed a survey questionnaire with three sections. The first section included demographic data such as gender, age, and site, level of education (e.g. postgraduate education in emergency nursing), years of experience in emergency nursing, and years of experience in triage with the ESI. The second section consisted of the 30 case scenarios from the ESI handbook. The third section of the survey questionnaire consisted of two questions addressing the level of nurses’ subjective confidence in applying the ESI accurately, including 1) “After assessing 30 triage case scenarios with the ESI instrument, how confident are you that your acuity ratings are correct?” and 2) “When applying the ESI in every day practice how confident are you about the accuracy of your triage decisions?”

At each study site, all participants completed the questionnaire individually while their group occupied a single room. The time limit was 1 h. Collaboration between study subjects was prohibited.

For each of the 30 case scenarios, individual participants were instructed to assign an ESI level. While completing the questionnaire and test, participants were allowed to consult a printed copy of the ESI algorithm and corresponding notes. These contain examples of external resources such as X-ray, labs, electrocardiogram, IV fluids, IV medications or specialist consultation or procedures. All collected data were manually entered into a spreadsheet of the SPSS statistics software® (Version 20).

### Data analysis

Descriptive statistics (means, frequencies and medians depending on level of measurement and data distribution) are presented to describe the socio-demographic characteristics of the participating triage nurses.

The rate of accuracy was calculated by the mean percentage of correct responses for the examples of each ESI category and scenario type (trauma, non-trauma, pediatric). As in a real triage environment, we defined incorrect ESI assignments to a triage category indicating higher acuity as “overtriage”, and inaccurate ESI assignments to a lower triage category as “undertriage”, respectively. The rates of overtriage and undertriage in case scenario ratings were expressed in percentages.

To measure interrater reliability for the ordinal data of the ESI, the coefficient, Krippendorff’s alpha was calculated. Krippendorff’s alpha applies to any number of raters; any number of categories, scale values or measures; any metric or level of measurement (nominal, ordinal); incomplete or missing data; and large and small sample sizes alike, with no minimum number of participants required [15]. The range of Krippendorff’s alpha is: 1 ≥ α ≥ 0; i.e., perfect agreement between raters is expressed with α = 1 and total disagreement between raters results in α = 0.

The nurses’ subjective confidence in applying the ESI was calculated by the mean percentage of response categories. This was done separately for the written test and clinical practice.

Significance level was established at a *p*-value of less than α = 0.05. All data were analyzed using IBM SPSS for Windows (20.0).

Two experts in the ESI triage process, an emergency physician and an advanced practice nurse, analyzed the case scenarios in which less than 75 % of the nurses’ ratings were correct. Possible reasons for inadequate triage level assignment were analyzed qualitatively and categorized using the decision points of the ESI algorithm as framework.

## Results

### Subjects

**Fig. 1:**
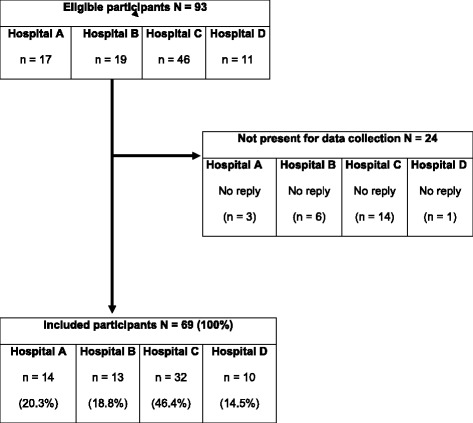
Recruitment of study participants

Subjects included 69 (65 females) of the 93 potentially eligible triage nurses from the four hospital emergency departments. All 69 were present on the days of data collection and completed the questionnaire (response rate: 74.2 %, Fig. 1). Nurses ages ranged from 25 to 60 years (mean 41.8, median 42), with 1–27 years of nursing experience (mean 9.8, median 10) and 2–48 months of triage experience (mean 17.5, median 12). Most of the triage nurses held a postgraduate diploma including emergency care (*n* = 45), intensive care (*n* = 8), anesthesia care (*n* = 3), or another degree such as a paramedic degree (*n* = 3). Fourteen of the triage nurses had no postgraduate diploma. Four triage nurses had two postgraduate diplomas.

### Accuracy

Table 2 summarizes nurses’ accuracy, stratified by ESI level. Nurses rated 59.6 % of cases correctly; with an under-triage rate of 26.8 % and an over-triage rate of 13.6 %. One answer out of 2070 case scenario ratings was missing. Overall, no significant differences between the four hospitals have been observed for rating the ESI correctly (*x*^2^ = 3.88; df = 3; *p* = 0.27).

**Table 2:** Accuracy of ESI-level assignment in four Swiss hospitals

	Correct Triage No. (%)	Undertriage No. (%)	Overtriage No. (%)	Chisq^b^	df	*p*-value
				*X* ^2^ =		
ESI Overall	1234 (59.6)	554 (26.8)	281 (13.6)	3.88	3	0.27
Hospital A	266 (63.3)	95 (22.6)	58 (13.8)	^a^		
Hospital B	228 (58.5)	122 (31.3)	40 (10.3)			
Hospital C	557 (58.0)	243 (25.3)	160 (16.7)			
Hospital D	183 (61.0)	94 (31.3)	23 (7.7)			
ESI 1 Total	177 (42.8)	237 (57.2)		38.48	3	0.00
ESI 1 Hospital A	45 (53.6)	39 (46.4)				
ESI 1 Hospital B	11 (14.1)	67 (85.9)				
ESI 1 Hospital C	100 (52.1)	92 (47.9)				
ESI 1 Hospital D	21 (35.0)	39 (65.0)				
ESI 2 Total	198 (47.8)	208 (50.2)	7 (1.7)	2.11	3	0.55
ESI 2 Hospital A	39 (46.4)	44 (52.4)	0 (0.0)	^a^		
ESI 2 Hospital B	42 (53.6)	36 (46.2)	0 (0.0)			
ESI 2 Hospital C	92 (47.9)	94 (49.0)	6 (3.1)			
ESI 2 Hospital D	25 (41.7)	34 (56.7)	1 (1.7)			
ESI 3 Total	351 (72.7)	61 (12.6)	71 (14.7)	4.92	3	0.18
ESI 3 Hospital A	77 (78.6)	9 (9.2)	12 (12.2)			
ESI 3 Hospital B	61 (67.0)	8 (8.8)	22 (24.2)			
ESI 3 Hospital C	158 (70.5)	36 (16.1)	30 (13.4)			
ESI 3 Hospital D	55 (78.6)	8 (11.4)	7 (10.0)			
ESI 4 Total	290 (70.0)	48 (11.6)	76 (18.4)	7.11	3	0.07
ESI 4 Hospital A	63 (75.0)	3 (3.6)	18 (21.4)			
ESI 4 Hospital B	62 (79.5)	11 (14.1)	5 (6.4)			
ESI 4 Hospital C	124 (64.6)	21 (10.9)	47 (24.5)			
ESI 4 Hospital D	41 (68.3)	13 (21.7)	6 (10.0)			
ESI 5 Total	218 (63.2)		127 (36.8)	24.61	3	0.00
ESI 5 Hospital a	42 (60.0)		28 (40.0)			
ESI 5 Hospital b	52 (80.0)		13 (20.0)			
ESI 5 Hospital c	83 (51.9)		77 (48.1)			
ESI 5 Hospital d	41 (82.0)		9 (18.0)			

Nurses’ accuracy overall, per hospital, and stratified by ESI level when rating case scenarios provided in the ESI implementation handbook. ESI 1—lifesaving intervention required, ESI 2—high risk situation, or confused/lethargic/disoriented, or severe pain/distress, ESI 3 more than one resource needed but vital signs within predefined limits (no danger zone vitals), ESI 4—1 resource needed, ESI 5—no resource needed

^a^ One answer is missing

^b^Test compared correct versus incorrect frequencies

When analyzing accuracy by triage level, 42.8 % of case scenario ratings on ESI level 1 were correct, with the correct assignment of ESI level 1 varying widely between sites (*x*^2^ = 38.48; df = 3; *p* < 0.001). ESI 1 ratings were more likely to be correct at the tertiary care hospitals, at which ESI refresher training was regularly provided. Case scenarios which included severe respiratory distress were inadequately assessed by most of the participants. One scenario involving intoxication combined with hypoventilation (No 30) was assessed correctly by only 17.4 % of the participants. A scenario including chronic COPD, shortness of breath and fever was correctly assessed by one (1.4 %) participant. (No 6).

Regarding ESI level 2, about half (50.2 %) of the case scenario ratings were incorrect. Overall, the six individual ESI level 2 case scenarios were assessed correctly by between 10.1 and 73.9 % of the participants. One case scenario, describing an accident case involving alcohol consumption (No. 7) was correctly assessed by 17 (24.6 %). Another case scenario regarding a baby with a fever (No 24) was correctly assessed by only seven (10.1 %) nurses.

Regarding ESI levels 3 and 4, more than 70 % of the case scenario ratings were correct (see Table 2). No significant differences were found between the four hospitals on ESI levels 2 through 4, whereas significant differences were detected on ESI level 5. For ESI level 5, triage nurses from the secondary care hospitals scored the case scenarios more accurately than triage nurses from the tertiary care hospitals (*x*^2^ = 24.6 %; df = 3; *p* < 0.001).

One case scenario (ESI level 5, child with fever, No 24) was correctly assessed by all participants. Twenty case scenarios were assessed correctly by more than 50 % of participants, whereas ten case scenarios were assessed correctly by less than 50 % of participants.

Regarding the scenario type, 58 % of the pediatric cases scenarios, 58 % of the non-trauma, and 64 % of the trauma case scenarios (*x*^2^ = 6.45 %; df = 2; *p* = 0.04) were correctly assessed by the participating triage nurses (see Table 3). Postgraduate education, years of ED nursing experience or years of triage experience had no significant effect on accuracy (see Table 4).

**Table 3:** Accuracy according to scenario type

	Correct Triage No. (%)	Undertriage No. (%)	Overtriage No. (%)	Chisq^b^	df	*p*-value
				*X* ^2^ =		
ESI Overall	1234 (59.6)	554 (26.8)	281 (13.6)	6.45	2	0.04
Trauma	396 (63.8)	168 (27.0)	57 (9.2)			
Non-trauma	596 (57.6)	320 (31.0)	118 (11.4)	^a^		
Pediatrics	242 (58.4)	66 (16.0)	106 (25.6)			

Nurses’ accuracy overall, per scenario type, when rating case scenarios provided in the ESI implementation handbook. Pediatric was defined as scenarios with patients aged younger than 16 (including both trauma and non-trauma)

^a^One answer is missing

^b^Test compared correct versus incorrect frequencies

**Table 4:** Relationship of triage nurse characteristics and correct triage

Characteristics of triage nurses (*N* = 69)	Correct Triage No. (%)	Chisqu.	df	*p*-value
Postgraduate degree		0.09	1	0.76
Without	248 (59.1 %)			
With	986 (59.8 %)			
ED nursing experience		0.28	1	0.6
≤ 5 years	363 (60.5 %)			
> 5 years	871 (59.3 %)			
Triage experience		5.58	3	0.13
0–12 months	610 (58.1)			
13–24 months	302 (59.2)			
25–36 months	201 (60.9)			
37–48 months	121 (67.2)			

Level of education (e.g. postgraduate education in emergency nursing), years of experience in emergency nursing, and years of experience in triage with the ESI is presented

### Interrater reliability

Overall, interrater reliability of triage nurses was 0.78 (Krippendorff’s alpha). Triage nurses’ inter-rater reliability in the individual sites was slightly higher than the overall inter-rater reliability (Table 5).

**Table 5:** Interrater reliability of triage nurses in four different hospitals

	Krippendorff’s α = (95 % CI)	N	Units
Total	0.78 (0.74–0.81)	69	30
Hospital A	0.78 (0.74–0.82)	14	30
Hospital B	0.80 (0.72–0.84)	13	30
Hospital C	0.79 (0.76–0.82)	32	30
Hospital D	0.84 (0.81–0.86)	10	30

For the interrater reliability Krippendorff’s α is presented. Perfect agreement between raters would result in α = 1, and total disagreement between raters in α = 0

### Subjective confidence

Triage nurses’ subjective confidence in applying the ESI in the written case scenarios was high. “Confident” or “very confident” ratings occurred from 54 nurses (78 %). No nurse felt “very unconfident”. Regarding applying the ESI in their daily clinical practice, 85.5 % (59 nurses) gave “very confident” or “confident” ratings. No nurse felt very unconfident.

## Discussion

Applying written patient case scenarios from the ESI handbook for triage competency testing in four Swiss EDs resulted in low accuracy, despite good inter-rater reliability and high confidence of triage nurses that they could correctly use the ESI.

Accuracy of nurses’ scoring of case scenarios for ESI level 1 and level 2 were rated noticeably lower than the average. On these ESI levels, less than half of the test ratings were accurate. The most significant differences among the four hospitals were observed at ESI level 1. Regarding setting-related influences on accuracy, the particularly poor performance in triage level assignments of the triage nurses in Hospital B as well as the wide range of case scenario rating results among triage nurses of all four hospitals is obvious. The very low percentage correct for two of the questions suggests that these test questions might be particularly challenging or may be ambiguous. In clinical practice, usually ESI level 1 corresponds to the smallest group of patients [7]. Regarding the individual nurses and the settings, triage nurses may have less practical experience in triaging ESI level 1 patients, which might contribute to explaining their underperformance in these case scenario ratings. The significantly higher accuracy in ESI-level 1 case scenarios in tertiary care hospitals compared to secondary care hospitals supports this assumption, since it is expected that tertiary hospitals are more regularly confronted with ESI level 1 patients. Variations regarding the accuracy among the four hospitals might further depend on variability of training programs for nurses and case mix variations, e.g. in some EDs only adult patients are treated. Finally, as regards training, the tertiary hospitals at which the nurses performed better regarding ESI 1 patients had regular refresher training, whereas the nurses at the secondary hospitals did not.

In contrast, ESI level 3 corresponds to a very large group of ED patients [7]. This might explain the highest accuracy in ESI level 3 ratings, as triage nurses are confronted to these situations more frequently. The significant differences between tertiary and secondary hospitals on correctly rating patients at ESI level 5 could reflect the assimilation of triage nurses to the setting. Nurses at secondary hospitals would be more likely to treat ESI level 5 patients more frequently.

In another single center study [16] performed in the USA, accuracy of acuity rating using pediatric case scenarios adapted from the ESI implementation handbook showed higher accuracy than was achieved by the nurses in our study. However, the data collection in that study occurred immediately after the first ESI training session. Therefore, their higher accuracy may have been enabled by adaption of the original case scenarios and by the provision of training immediately before testing. In contrast, nurses in our study did not receive any refresher training immediately before data were collected. Further, it is not known exactly how standardized case scenarios were adapted for use as the 20 pediatric patient scenarios, making direct comparison between these studies more problematic.

Reasons for frequently inaccurate determination of triage acuity in our study cannot be determined with certainty. However, several factors might contribute. A lack of nurses’ factual knowledge is likely the most important factor. Also, having only infrequent exposure to certain clinical situations (e.g. treatment of pediatric patients) might contribute. Further, the setting might play a role. The time gap between training and testing may have had a large effect. Further, we identified specific protocols in some hospitals that could be the reason for inaccurate triage level assignment. For example, a blood sample is mandatory before a psychiatric referral in at least one hospital (case No 11).

Only for one case scenario (Case Number 1) do we believe the translation might have cause an incorrect triage level; specifically for the case involving gastrointestinal bleeding, due to the translation of the word for the color of the patient’s stool. When translated literally, this expression is used to describe normal stool color in German. Surprisingly, this mistake was not prevented with the guideline based translation approach. Finally, case-scenarios might contribute by not providing enough information on the patients’ situation, for example the description of a wound (case No 4). Two cases might be ambiguous and therefore the gold standard not to be conclusive: In case No 6 and No 30 the need of an *immediate* life-saving intervention (decision point A) may be questionable. The information could have been written more clearly for these two cases. Further, the written case scenarios may not adequately reflect a real triage environment as experienced in daily clinical practice. Case scenarios usually provide a short, potentially ambiguous description of a clinical situation, to which the reader is at risk of making incorrect inferences and interpretations. Studies that were performed in real triage environments such as the retrospectively double checked triage nurse assessments by a triage expert showed an accuracy between 77 and 94 % [3, 4, 7]. The advantage of written case scenarios for triage competency testing, however, is that test conditions can be standardized and confounding factors of a real triage environment such as ED crowding can be excluded.

Factual knowledge has been found to be more important than years of emergency nursing or triage experience to predict triage decision accuracy [11]. The results of our study concur with this prior finding by showing that there was no discernable influence of postgraduate training, years of nursing experience, or years of triage experience upon accuracy of ESI scoring (see Table 4).

The inter-rater reliability among triage nurses in our study was high considering international standards. Acceptable inter-rater reliability levels varies between .67 and .80, respectively [17–20], and among multiple assessors a level higher than .74 is considered to be excellent [21]. Two differing explanations for high inter-rater reliability are plausible: It is possible that most triage nurses’ training is sufficiently similar, such that nurses in the different settings would make the same mistakes, as compared to the gold standard. Second, in the light of moderate accuracy, the cases might be written without optimum clarity, such that the accuracy of the test items could be questioned. With respect to the case scenarios provided by the implementation handbook version 2005, we found that some of the case scenarios do not provide sufficient information to eliminate ambiguity in the assignment of a distinct triage level. The scenarios seem suitable for initiating discussions on them, which would be useful for training purposes. However, for competency testing, case scenarios should be unambiguous.

It is a troubling finding that nurses’ subjective confidence in their ability to correctly apply the ESI triage instrument exceeded their ability to rate scenarios correctly. Nurses’ confidence was very high (85.5 %) with regard to ESI application during daily clinical practice, and high (78 %) when rating the written case scenarios. In the study of Singer et al. [22], the majority (93.4 %) of participants was very satisfied or satisfied with the ESI. Satisfaction referred to practicability in applying the ESI algorithm, especially for less experienced triage nurses. Durani et al. [16] examined the level of comfort with the ESI algorithm and found 58 % of triage nurses who felt very comfortable or comfortable using the ESI. However, it may also be that nurses overestimate their ability to make accurate triage decisions. This may in part explain undertriage in real triage environments when applying the ESI [7, 23, 24].

Flawed self-assessment of one’s own abilities is usually due to one of the following reasons [25]. First, erroneous self-assessments can arise because assessors may not have all of the information necessary to provide accurate assessments. Raters cannot take into account what they do not know. Second, erroneous self-assessments arise because people neglect relevant and useful information that they do have in hand [25]. Flawed self-assessment is a widespread multidisciplinary phenomenon that occurs among professionals of different hierarchical levels [25–27].

Another possible explanation for the demonstrated moderate accuracy may be that written case scenarios may not be the ideal method for a standardized assessment of ED nurses’ accuracy of rating a patient’s clinical acuity. A written case scenario may not be realistic enough for the nurses to implement what is needed for the scenario.

For triage training, the combination of multiple teaching methods and training approaches [12] such as human patient simulation, computer learning games or virtual reality triage training [12, 28–30] might offer an alternative or adjunct for triage training in non-clinical situations. However, these would be much more labor-intensive than methods currently used to train nurses in most locations, and the cost-benefit ratio of such training can only be conjectured. Potential options to improve triage skills in clinical practice include regular refresher training programs in ESI application, testing of factual knowledge (e.g. with case scenarios), and direct observation of triage performance including feedback.

### Limitations

This study has several limitations to consider. Nurses were aware of the study’s purpose, and the simultaneous testing of nurses in a single room might have had an impact on nurses’ performance.

Sixty-nine of 93 potentially eligible triage nurses participated in the study, raising the possibility of a selection bias to occur. However, selection bias (the possibility that only high performers participated) is highly unlikely. The 24 potentially-eligible nurses who did not participate were absent on the days of data collection because these nurses were not scheduled for duty on those days. One of the strengths of the study is the involvement of a very heterogeneous sample of 69 experienced triage nurses from four hospital EDs reflecting various professional backgrounds and experiences in different settings, confirming the reliability of the ESI when applied in written patient case scenarios.

Varying amounts of time elapsed between initial ESI training and ESI testing in the four institutions. Different results may have been obtained if nurses had been tested immediately after initial training of nurses was performed. If the training received by the nurses at the different hospitals differed markedly, such differences may confound the results. However, trainings in all institutions followed the recommendations of the ESI implementation handbook.

Our study was performed using case scenarios from handbook 2005. The latest edition of the handbook, version 2012, which was not yet available when performing the present study, provides more case scenarios which might have had an impact on our results. However, 29 of 30 scenarios are identical, one has been omitted in the current version [31].

## Conclusions

Written case scenarios may theoretically appear to be a feasible tool to examine nurses’ competence at accurately rating a patient’s acuity by applying the ESI across different settings in a standardized fashion. However, ESI competency testing scenarios, as currently written, frequently lead to patient acuity ratings that differ from the rating intended by the authors of these scenarios, despite a significant previous exposure of nurses to the ESI tool. As is true for any instrument used to test clinicians’ ability to determine a clinical rating, test questions should be assessed for performance criteria.
